# P-894. MRSA bacteremia with high MIC to Vancomycin: A comparison study of Daptomycin, Ceftaroline, and Both

**DOI:** 10.1093/ofid/ofae631.1085

**Published:** 2025-01-29

**Authors:** Zaid Al khouri, Anood Al Qura'an, Mollie VanNatta, Mohammad Alam, Ruhul Munshi, Alexandre Malek

**Affiliations:** Louisiana State University Health Sciences Center Shreveport, Shreveport, Louisiana; Louisiana State University Health Sciences Center Shreveport, Shreveport, Louisiana; Ochsner LSU Health Shreveport, Shreveport, Louisiana; Louisiana State University Health Sciences Center, Shreveport, LA, USA, shreveport, Louisiana; LSU Health Shreveport, Shreveport, Louisiana; Louisiana State University Health Sciences Center Shreveport, Shreveport, Louisiana

## Abstract

**Background:**

Daptomycin (DAP) and/or ceftaroline (CPT) have been used in pts with MRSA bacteremia (MRSAB) with minimum inhibitory concentration (MIC) 2 ug/ml to vancomycin (Vanc). However, head-to-head studies comparing DAP versus CPT or DAP-CPT combination are lacking. In this study, we sought to describe the outcomes and characteristics of pts with MRSAB who received DAP, CPT, or both.

**Figure:**
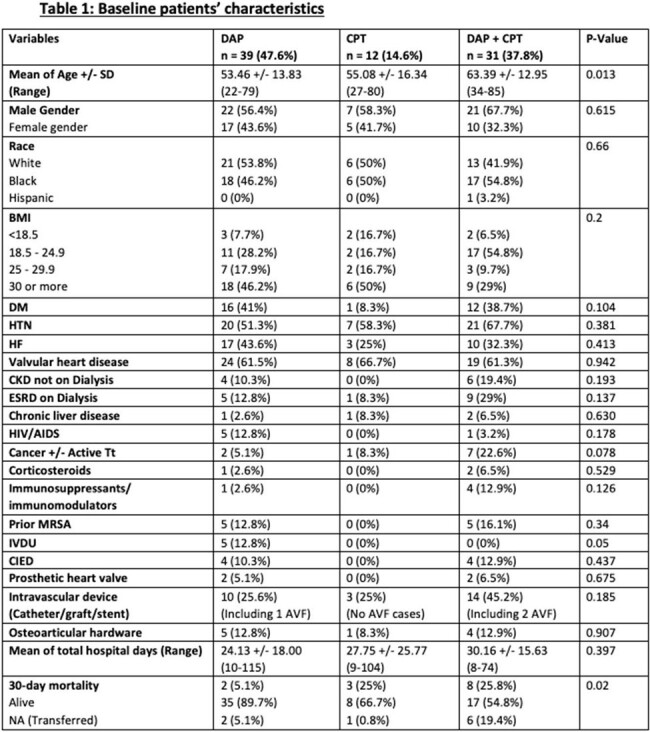

**Methods:**

We performed a retrospective cohort study of adult pts admitted to Ochsner LSU Health Shreveport– Academic Medical Center who were diagnosed with MRSAB MIC 2 ug/ml to Vanc and received DAP, CPT, or both for at least >=72 hours. Pts’ demographics, comorbidities, source of infection, and mortality data were all collected.

**Figure:**
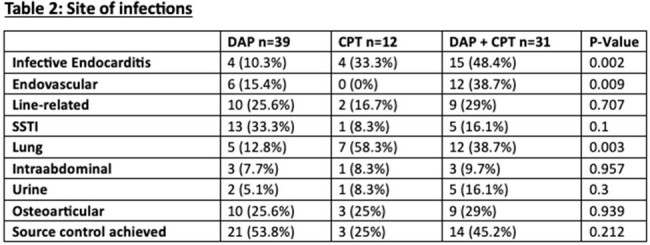

**Results:**

We identified 82 pts with MRSAB MIC 2 ug/ml to Vanc. We divided them into 3 groups: DAP (n = 39, 47.6%), CPT (n = 12, 14.6%), and DAP-CPT (n = 31, 37.8%). The mean age was 53 vs 55 vs 63 y, respectively (p-value=0.013). Males were more prevalent than females in the 3 groups. Pts’ demographics at baseline were comparable between the three groups, and there were no statistically significant differences between the three groups in terms of comorbidities. Prior MRSA bacteremia was reported in 5 pts in the DAP-CPT group and DAP group (p-value=0.34). IV drug use was only reported in 5 pts who received DAP(p-value=0.05). SSTI (33.3%) was the leading cause of bacteremia in DAP group followed by osteoarticular and CVC-related infections in 25.6% each. The lung was the most source of infection in 58.3% of Pts in CPT group. In the DAP-CPT arm, IE was the main source of infection (48.4%), followed by endovascular and lung in 38.7% each. IE was statistically significant in DAP-CPT group (p-value=0.002). The mean of MRSA bacteremia was 4 vs 5 vs 9 days in DAP, CPT, and DAP-CPT, respectively while the clearance of MRSA bacteremia after the initiation of each regimen was faster and in favor of Pts who received DAP-CPT (2.3 d), p-value< 0.009. However, the 30-day mortality was higher in patients of the DAP-CPT group (25.8%), p-value=0.02.

**Figure:**
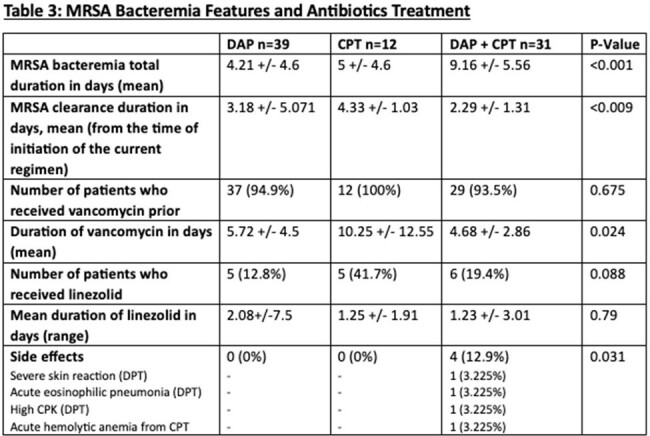

**Conclusion:**

Our data demonstrated that pts who received DAP-CPT combination therapy cleared MRSA bacteremia faster than those who received DAP or CPT as monotherapy. Further studies are needed to elucidate if early initiation of the DAP-CPT combination could reduce the mortality rate.

**Disclosures:**

**All Authors**: No reported disclosures

